# An economic evaluation of restorative justice post-sentence in England and Wales

**DOI:** 10.3389/fpsyg.2023.1162286

**Published:** 2023-11-16

**Authors:** Frank Grimsey Jones, Lucy Jaffé, Lucy Harris, Jon Franklin, Lisa Allam, Joanna Shapland

**Affiliations:** ^1^Why me? Transforming Lives Through Restorative Justice, London, United Kingdom; ^2^Pro Bono Economics, London, United Kingdom; ^3^Office of the Police and Crime Commissioner for Hampshire, Hampshire, United Kingdom; ^4^School of Law, University of Sheffield, Sheffield, United Kingdom

**Keywords:** restorative justice, economic evaluation, reoffending, criminal justice, restorative practice

## Abstract

**Introduction:**

Participation in restorative justice interventions post-sentence has been shown to reduce reoffending and mitigate harm to victims. Investment in, and access to, restorative justice remains limited in England and Wales. An economic model was developed to synthesize the available evidence in order to develop contemporary and robust estimates of the economic impact of investment in restorative justice interventions.

**Methods:**

This research focused on direct and indirect restorative justice interventions for victims and offenders post-sentence in England and Wales. Included offences were those with an identifiable victim. A model was developed to estimate the social benefit–cost ratio of restorative justice, as well as the direct financial return to the criminal justice system. The modeled benefits of restorative justice included reductions in reoffending and direct wellbeing benefits for victims. It was not possible to incorporate direct wellbeing benefits for offenders due to evidence gaps.

**Results:**

In the model, 8% of referrals to restorative justice resulted in direct restorative justice interventions and 19% resulted in indirect Restorative justice interventions. The modeled cost of the restorative justice pathway per direct intervention was £3,394. The base case estimate for the social benefit–cost ratio of restorative justice was £14 per £1 invested, with a direct return to the criminal justice system of £4 as a result of substantial reductions in reoffending. Scenario analysis suggested a plausible range of £7 to £20 social benefit per £1 invested. Hypothetically, increasing the proportion of eligible cases referred for a restorative justice intervention from 15 to 40% could be associated with an increase in investment of £5 m, and benefits to the criminal justice system totaling £22 m, implying a net saving of £17 m.

**Conclusion:**

The research suggests that Restorative justice has the potential to yield a substantial social return on investment (SROI) and direct return on investment to the criminal justice system. The economic case for investment in restorative justice centers on identifying offenders with a high risk of offending and enabling them to participate in an intervention that has been repeatedly demonstrated to help them to change their behavior.

## 1 Introduction

Restorative justice allows people affected by crime to communicate with the person responsible. It gives an opportunity to discuss what happened and explore how people have been affected. This is often achieved via a face-to-face meeting. Victims can explain how it has impacted them, ask questions and give the offender the opportunity to respond, seek assurances that it will not happen again, and have a say in how the harm can be repaired. Restorative justice is voluntary for both victim and offender.

This research adopts the commonly used definition of restorative justice, that it is ‘a process whereby parties with a stake in a specific offence collectively resolve how to deal with the aftermath of the offence and its implications for the future’ (Marshall, 1999).

A major restorative justice trial in England and Wales found that 85% of victims who participate in restorative justice are satisfied with the experience (Shapland et al., 2007). A literature review and meta-analysis showed that restorative justice can reduce reoffending and benefit victims and offenders (Strang et al., 2013).

The Victims’ Code of Practice states that all victims of crime are entitled to information about restorative justice (Ministry of Justice, 2020). But only 5% of victims with a known offender recall being told about this (Office for National Statistics, 2019).

Around a quarter of proven offenders are proven to reoffend within a year, with an average of three to four proven reoffences per reoffender (Ministry of Justice, 2022). For theft offences, this rises to half of proven offenders reoffending within a year, with approximately five proven reoffences per reoffender (Ministry of Justice, 2022). This represents only a subset of reoffending. This cohort commit a substantially higher number of reoffences that do not result in a proven outcome, so do not appear within Government statistics. Home Office research estimated that in 2016 the total economic and social costs of reoffending in the first year were £18 billion (Newton et al., 2019). This research excluded the impact of reoffending on offenders themselves. Imprisonment as a result of reoffending has a substantial impact on offenders themselves and their families. Interventions that help to break the cycle of reoffending have the potential to result in economic benefits that exceed their costs, as well as improving the welfare of some of the most disadvantaged in society.

There have been few rigorous economic evaluations of these interventions direct restorative justice interventions. Previous economic evaluations have been limited by the fact that they are predominantly trial-based and evaluate restorative justice delivered to a specific group of people, in a specific way, at a specific time (Shapland et al., 2008; Matrix Evidence, 2009; Furman, 2012). This economic evaluation presents the results of a cost-social benefit model comparing conventional justice supplemented with a restorative justice intervention, to conventional justice without a restorative justice intervention. It provides a framework for the economic evaluation of restorative justice that has the flexibility to model a range of scenarios and can be updated to incorporate new evidence.

The purpose of this analysis was to evaluate the economic impact of investing in restorative justice, as a supplement to conventional justice, in England and Wales. It focuses on restorative justice interventions taking place following a criminal offence with a proven outcome. A report summarizing the research, a copy of the economic model and a number of explanatory videos, are available on the Why me? website (Grimsey Jones and Harris, 2022).

## 2 Methods

### 2.1 Evidence review

We conducted a rapid review of relevant literature to ensure the modelling approach, and inputs used, were informed by the best available evidence. The review was conducted using internet search engines and the following databases: Restorative Justice Council website, What Works in Policing website and the Gov.uk website (College of Policing, 2020; Restorative Justice Council, 2020). The review results were supplemented by engaging with relevant stakeholders from across the restorative justice sector. Insights were synthesized by one reviewer, then validated by a second reviewer.

Existing economic evaluations of restorative justice can be divided into those where restorative justice is a supplement to conventional justice and those where restorative justice is a substitute for conventional justice. These two groups of studies focus on different populations and different potential benefits of restorative justice. Studies of restorative justice as a supplement, focus on the benefits of restorative justice in reducing reoffending amongst those who have committed relatively more serious crimes. Studies of restorative justice as a substitute, focus on whether the costs of delivering restorative justice offer a cheaper alternative to implementing conventional justice responses to crime for lower-level offenders.

Previous economic evaluations of restorative justice have multiple methodological limitations. The most important limitations are in the methods applied to estimate the impact of restorative justice on reoffending, and the costs of the conventional justice process (Macdonald et al., 2017). There are only a couple of studies that use estimates for the impact of restorative justice on reoffending taken from high-quality studies with a low risk of bias (Shapland et al., 2008; Matrix Evidence, 2009). There are additional limitations that apply across all studies. They are generally old, and the assumptions made may no longer be relevant (Shapland et al., 2004). They apply very limited use of sensitivity analysis, meaning that the robustness of findings to different assumptions is unknown. Some of the key studies are policy reports, with limited peer-reviewed research on the economic impact of restorative justice.

### 2.2 Data collection

There is limited availability of high-quality national data on restorative justice provision. We therefore gathered data from three English police force areas, one youth offending service and one independent restorative justice provider. These data included information on the population accessing restorative justice, rates of attrition and rates of resource use throughout the restorative justice pathway. Interviews were conducted to validate the data and ensure like-for-like comparison. The face validity and relevance were used to determine which data to implemented in the model. Henceforth the data sources are referred to as:

- Police Force Area 1
- Police Force Area 2
- Police Force Area 3
- Youth Offending Service 1
- Restorative Justice Provider 1

### 2.3 Model structure

The Manning Cost–Benefit Tool (MCBT) is a published and validated economic model in Microsoft Excel for evaluating the economic impact of interventions to reduce crime (Manning et al., 2019). We used the MCBT as a starting point to develop an economic model as part of this study. The adapted model followed a decision tree structure. Deterministic sensitivity analysis and subgroup analysis were used to model the sensitivity of the results to uncertainty and model hypothetical scenarios. A societal perspective was adopted, in which all relevant costs and benefits were modeled, where there was sufficient evidence to do so. A governmental perspective was used within sensitivity analysis. We used 2021 costs, which were inflated where necessary, and a discount rate of 3.5% (HM Treasury, 2022).

**Figure 1:**
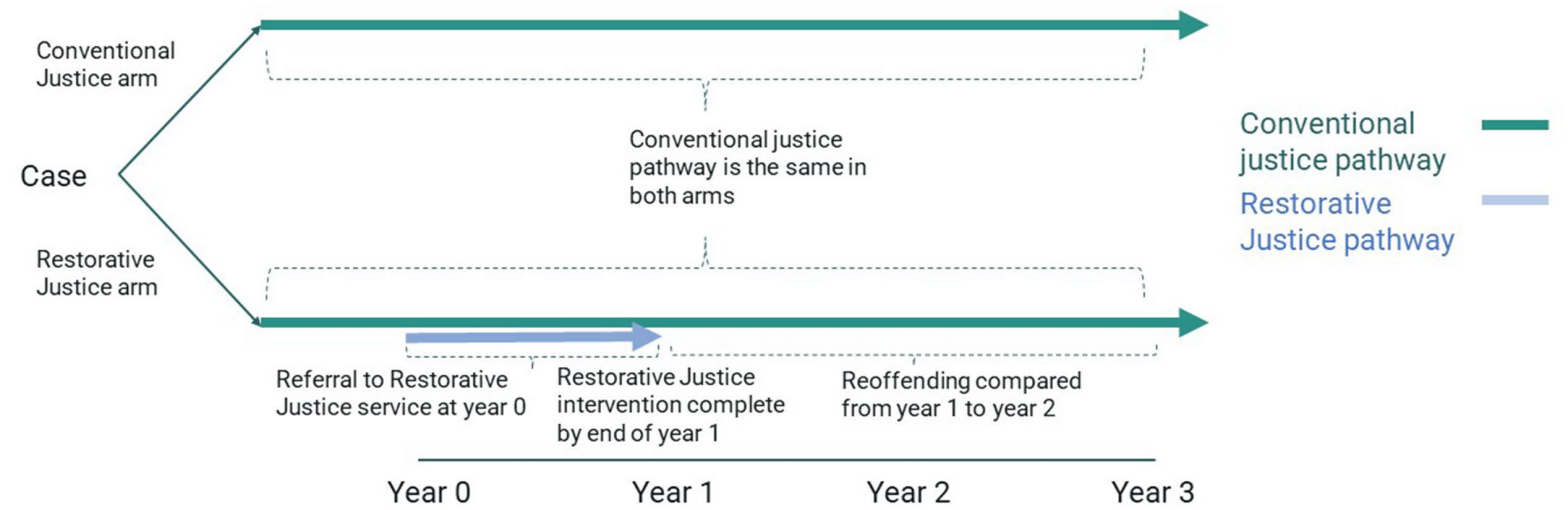
Schematic of the impacts of the conventional justice and restorative justice Pathways. Source: Grimsey Jones and Harris (2022).

Figure 1 illustrates the model structure. The model was separated into one-year periods. Each victim-offender grouping (henceforth termed a ‘case’) entered either the conventional justice arm or the restorative justice arm (which consists of the conventional justice pathway, supplemented by a restorative justice intervention). It had a two-year time horizon in the base case. The delivery of a restorative justice intervention was modeled over a one-year period, with the impact of the intervention modeled over a subsequent one-year period. Extended time horizons were tested as part of sensitivity analysis.

Each case in the restorative justice arm entered the restorative justice pathway in the first year. The pathway had a number of stages:

- Referral: the restorative justice service receives a request to deliver restorative justice to a particular case. It can come from the victim or the offender, or a service working with the victim or offender.
- Assessment and Consent: the restorative justice service determines whether the case is suitable for a restorative justice intervention and gains consent from the victim and offender. In practice, this stage can comprise a range of different visits, phone calls and/or emails.
- Intervention: the victim and offender participate in a restorative justice intervention. This can either be direct (the victim and offender meet) or indirect (the victim and offender communicate with each other but do not meet).

Some cases drop out from the pathway at the ‘Referral’ and ‘Assessment and Consent’ stages. In the base case we assumed that these cases were associated with the costs incurred at these stages but no benefits. Cases that progressed to a restorative justice intervention, were associated with costs, and benefits. Benefits were grouped according to the recipient. They included direct impacts on the wellbeing of the victim (Section 2.7.2) and the impact of reduced reoffending (Section 2.7.1). It was not possible to model direct impacts on the wellbeing of the offender (Section 2.7.3). Costs were applied in the first year of the model and benefits were applied in the second year of the model in the base and extended in sensitivity analysis.

### 2.4 Population

#### 2.4.1 Victims

We included all victims in the analysis. Experts, including researchers and frontline practitioners, advised that the effectiveness of restorative justice is independent of the characteristics of victims. This was aligned with the published literature, as we did not find suitable data to suggest a relationship between victim characteristics and the effectiveness of restorative justice. The Shapland reports do not state whether there was a significant relationship between victim demographics and the effectiveness of restorative justice (Shapland et al., 2008). It does state that a number of variables related to victims, were not found to be statistically significantly linked to the effectiveness of restorative justice. These included: ‘victim views about the conference, whether the victim and offender knew each other, whether victims accepted any apology the offender made, or whether victims thought the offender was sincere.’

Hence, the effects of restorative justice on victims were assumed to be the same for all victims regardless of their characteristics.

#### 2.4.2 Offenders

We included all offenders who committed a crime with a known victim. Table 1 presents national data on proven crimes (Flatley, 2021). This was used as the mix of index offences in the economic model. The average age for offenders was assumed to be 31, informed by data from Police Force Area 2. It was assumed that the baseline reoffending rate was dependent on offence types, but the effectiveness of restorative justice was not. Ministry of Justice data shows that the rate of reoffending, and type of reoffending, are closely linked to index office (Ministry of Justice, 2022). The fourth Shapland report did not find a statistically significant relationship between the effectiveness of restorative justice and any demographic characteristics of offenders, or their index offence (Shapland et al., 2008). The Strang et al. (2013) meta-analysis presents some inconclusive evidence as to whether the effectiveness of restorative justice is linked to index offence.

**Table 1:** Cases by offence type.

Offence type	Offence subtype	Percentage of cases
Violence against the person	Homicide	0.03%
	Violence with injury	22.11%
	Violence without injury	28.59%
Sexual	Rape	0.75%
	Other sexual offences	1.44%
Robbery	Robbery	1.94%
	Commercial robbery	0.00%
Theft	Domestic burglary	6.91%
	Commercial burglary	0.00%
	Commercial theft	0.00%
	Theft of vehicle	2.90%
	Theft of commercial vehicle	0.00%
	Theft from vehicle	7.96%
	Theft from commercial vehicle	0.00%
	Theft from person	2.76%
Criminal damage and arson	Criminal damage arson	1.01%
	Criminal damage other	23.61%
	Commercial criminal damage–arson	0.00%
	Commercial criminal damage–other	0.00%

The costs and effects of delivering a restorative justice intervention were assumed to be independent of offence type and other offender characteristics. Experts advised that this was an appropriate assumption and it is supported by previous research (Shapland et al., 2008).

### 2.5 Intervention

Restorative justice interventions can either be direct or indirect. Direct restorative justice interventions are where the victim and offender meet. Indirect interventions are where the victim and offender engage in two-way communication, but do not meet in person, with the support of an independent facilitator. This includes the exchange of letters or emails.

**Figure 2:**
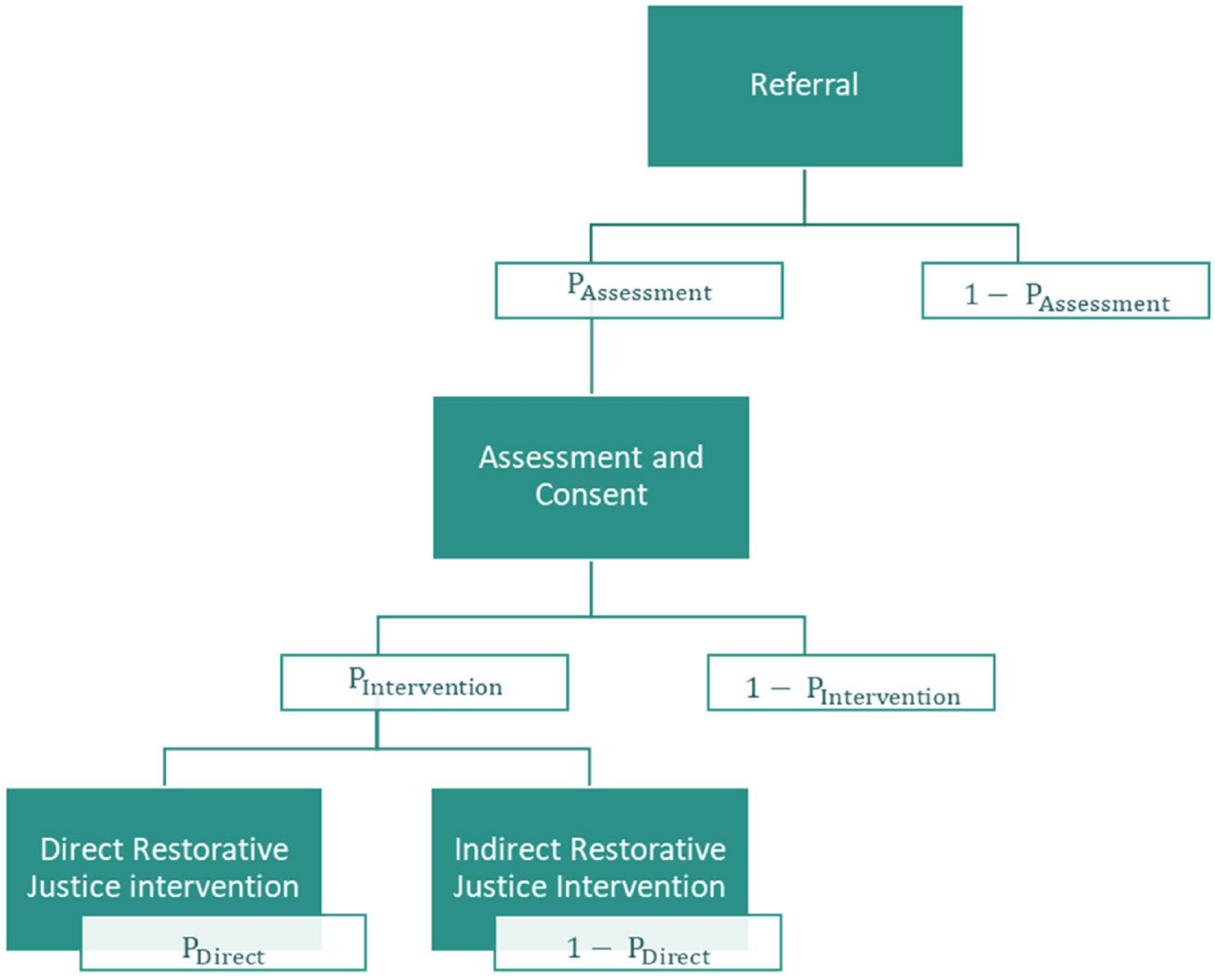
Rate of attrition as cases progress through the restorative justice pathway. Source: Grimsey Jones and Harris (2022).

This research assumed that restorative justice was initiated via a referral and receipt of consent from the victim or offender, post-sentence (Figure 2). Once a referral was made, an assessment was conducted to determine whether the case was suitable, and consent was sought from both the victim and the offender. Various estimates for the rate of attrition were gathered and are presented in Supplementary material. The estimates from Police Force Area 2 were implemented in the base case as the source appeared more reliable and the estimates represent a reasonable midpoint of the other available estimates (Table 2).

**Table 2:** Rates of attrition during the restorative justice pathway.

Stage	Value	Source
Cases that drop out at the Referral stage	41%	Police Force Area 2, 2021
Cases that drop out at the Assessment and Consent stage	40%	Police Force Area 2, 2021
Cases that engage in an indirect intervention	6%	Police Force Area 2, 2021
Cases that engage in a direct intervention	13%	Police Force Area 2, 2021

### 2.6 Comparator

As discussed in Section 2.2, different offenders can enter the restorative justice pathway at different points in the conventional justice pathway. This research models the impact of restorative justice interventions applied as a supplement to conventional justice, not a substitute for conventional justice. The group of offenders included have received relatively serious sentences that they must comply with, regardless of whether or not they participate in a restorative justice intervention. There are no services that these offenders or victims forgo as a result of participating in a restorative justice intervention. For this reason, we did not need to model the costs or benefits of the conventional justice process these offenders follow because this is not affected by the presence of restorative justice.

### 2.7 Outcomes

#### 2.7.1 Reoffending

##### 2.7.1.1 Reductions in proven reoffending

Reoffending rates were modeled by calculating a baseline reoffending rate, and then applying a treatment effect for cases that engaged in a restorative justice intervention.

Baseline reoffending rates were calculated using Government datasets (Ministry of Justice, 2022). We assumed that restorative justice interventions could only be implemented for crimes where there was an identifiable victim. Baseline reoffending rates vary depending on the index offence. They are relatively high for theft offences and relatively lower for sexual offences (Ministry of Justice, 2022). There is a strong serial correlation between offence types. For example, if an individual’s index offence is violence against the person and they reoffend, it is more likely that their reoffence is another violence against the person offence relative to individuals who commit different index offences (Ministry of Justice, 2022). This demonstrates the importance of modelling the expected reoffence type, conditional on the index offence type.

The Strang 2013 study includes a meta-analysis of the impact of restorative justice on the number of proven reoffences per case during a two-year follow-up period (Strang et al., 2013). The study is a high-quality source of evidence because it is a meta-analysis that only includes studies with rigorous methodologies, collected using a systematic search (Strang et al., 2013). The research employed an appropriate method for synthesizing the effects across the included studies.

There is a high level of heterogeneity among the studies included in Strang 2013 regarding the offender characteristics, the victim characteristics, the positioning within the conventional justice pathway and the region in which the studies took place (Strang et al., 2013). This is associated with benefits and limitations. The variation between the studies can be interpreted as yielding estimates with a high level of generalizability. This means that the study results could be interpreted as relevant to a wide range of restorative justice services in a wide range of settings. One limitation of this heterogeneity is that the results are an average and the effectiveness of restorative justice in practice may vary depending on the context. From a modelling perspective, it was challenging to align the inclusion criteria of the studies within Strang 2013, with some of the other available sources (Strang et al., 2013). For example, the mix of different index offence types included within the Strang 2013 meta-analysis is determined by the inclusion criteria of the included studies and may not be representative of the index offence types committed by offenders suitable for, or able to access, restorative justice in England and Wales.

The Strang results are presented as a standard mean difference, this was converted to a rate ratio using a method from the Cochrane Handbook (Table 3; Higgins and Green, 2011). The rate ratio from across all studies, 0.715, aligned with the pooled rate ratio reported in the fourth Shapland report for the JRC trials (Shapland et al., 2008). Close alignment was expected given that the JRC trials within the fourth Shapland report are a subgroup of trials included within Strang 2013. The rate ratio for the subgroup of studies where restorative justice was a supplement to conventional justice was lower (ratio of 0.705), implying a greater reducing in reoffending in this subgroup. We chose to use 0.715 in the base case with uncertainty tested within the scenario analysis.

**Table 3:** Transformation of recidivism reduction estimates to estimate the risk of reoffending.

Theme	Input	Value (CI)	Source
Strang 2013, all studies	Standard mean difference	−0.185 (−0.285 to −0.085)	Strang et al. (2013)
	Log rate ratio	−0.336 (−0.517 to −0.154)	Calculated using Cochrane Formula (Higgins and Green, 2011)
	Rate ratio	0.715 (0.596 to 0.857)	Calculated
Strang 2013, restorative justice as a supplement only	Standard mean difference	−0.193 (−0.297 to −0.089)	Strang et al. (2013)
	Log rate ratio	−0.350 (−0.539 to −0.161)	Calculated using Cochrane Formula (Higgins and Green, 2011)
	Rate ratio	0.705 (0.584 to 0.851)	Calculated
Shapland report	Rate ratio	0.715 (0.549 to 0.932)	Shapland et al. (2008)

The reoffending rate implemented in this table is pooled across offence types for illustrative purposes. In the context of the economic model, the method will be applied to each index offence type separately.

Whilst some experts suggested that restorative justice can reduce reoffending over the long term, we did find data to support this. Also, we were not able to access baseline reoffending rates after the first year. Consequently, restorative justice was assumed to impact reoffending for 1 year in the base case. Longer impacts were tested as part of sensitivity analysis.

##### 2.7.1.2 Reoffending multipliers

This research implemented a methodology developed by Pro-Bono Economics to use reductions in proven crime to estimate reductions in total crime (including crimes that are not proven) (Carr et al., 2020). The formulae for calculating the multipliers that connect proven crime with total crime are detailed below (Equation 1). These modeled relationships between proven crime and total crime, were applied to reductions in reoffending, to estimate reductions in total reoffending.

Equation 1: method for linking reductions in proven crime to total crime.


(1) Reduction in total crime=M1×M2×Reduction in proven crime

(2) Charged crime×M2=Total reported crime

(3) $$\begin{array}{c} \text{Charged crime}\times \text{Conviction ratio}\\ +\;\text{Other proven outcomes} \end{array}=\text{Total reported crime}$$

(4) Total reported crime×M1=Total crime

(5) Total reported crime×Reporting rate=Total crime

Where, for each type of crime,

Multiplier 1 (M1) is the ratio of crimes to reported crimes.

Multiplier 2 (M2) is the ratio of reported crimes to proven crimes.

Multiplier 1 is estimated within the Economic and Social Costs of Crime report, by comparing data on crimes reported to the police with data from the Crime Survey for England & Wales on total crime experienced within the population (Heeks et al., 2018). Table 4 presents estimates for Multiplier 1 for different offence types. There is wide variation, with almost all ‘Homicides’ being reported, but only a small fraction (<10%) of ‘Other Sexual Offences’, reported to the police. These data are subject to substantial uncertainty because some of the crimes reported within the Crime Survey for England & Wales may be a result of misperceptions about what constitutes a crime and there could be issues with misalignment in time periods.

**Table 4:** Crime multipliers by crime type, 2018.

Crime	Estimated total	Multiplier 1 (reported to total crime)
Homicide	570	1
Violence with injury	1,104,930	2.6
Violence without injury	852,900	1.5
Rape	121,750	3.4
Other sexual offences	1,137,320	16.5
Domestic Burglary	695,000	3.6
Theft of vehicle	68,000	0.8
Theft from vehicle	574,110	2.6
Theft from the person	459,240	5.9
Criminal damage – arson	22,600	1
Criminal damage – other	1,007,160	2
Commercial robbery	102,570	1
Commercial burglary	310,700	1
Commercial theft	8,400	1

Source: total number of crimes committed, PRCs and resultant multipliers (Heeks et al., 2018).

Estimating Multiplier 2, the relationship between reported crime and proven crime, is more complex. Proven crimes include convictions and a number of other proven crime outcomes, including out-of-court disposals. The method used to calculate Multiplier 2 is detailed in Equation 2.

Equation 2: calculating M2, the ratio of reported crimes to proven crimes.


(6) $$M2=\frac{\text{Proven reoffences}}{\text{Police recorded crime}}$$

(7) $$M2=\frac{\begin{array}{l} \left(\text{Number of cases charged}\;x\;\text{Conviction ratio}\right)\\ +\;\text{Other proven outcomes} \end{array}}{\text{Police reported crime}}$$

(8) $$\begin{array}{l} \text{Other proven outcomes}=\text{Out of court}\;\left(\text{formal}\right)\\ \;\;\;\;\;\;\;\;\;\;\;\;\;\;\;\;\;\;\;\;\;\;\;\;\;\;\;\;\;\;\;\;\;\;\;\;\;\;\;\;\;\;\;\;+\text{Out of court}\;\left(\text{informal}\right)\\ \;\;\;\;\;\;\;\;\;\;\;\;\;\;\;\;\;\;\;\;\;\;\;\;\;\;\;\;\;\;\;\;\;\;\;\;\;\;\;\;\;\;\;\;+\text{Taken into consideration}\\ \;\;\;\;\;\;\;\;\;\;\;\;\;\;\;\;\;\;\;\;\;\;\;\;\;\;\;\;\;\;\;\;\;\;\;\;\;\;\;\;\;\;\;\;+\text{Warning}\\ \;\;\;\;\;\;\;\;\;\;\;\;\;\;\;\;\;\;\;\;\;\;\;\;\;\;\;\;\;\;\;\;\;\;\;\;\;\;\;\;\;\;\;\;+\text{Penalty notice} \end{array}$$

Table 5 presents the proportion of offences resulting in each proven outcome, by offence type, as well as estimates for Multiplier 2. The estimates for Multiplier 2 seemed high at first glance, particularly for theft offences. Supplementary analysis was conducted to validate the estimates for Multiplier 2, which is reported in the supplement. Overall, there was a mixed picture as to whether the Multiplier 2 estimate for theft offences were plausible and they are subject to substantial uncertainty. Multiplier 2 was varied in sensitivity analysis to account for this uncertainty.

**Table 5:** Number of crimes by type and sentence type, 2020–2021.

	Violence against the person	Sexual	Robbery	Theft	Criminal damage and Arson
Charged/Summonsed	5.2%	2.9%	6.6%	4.2%	4.3%
Taken into consideration	0.0%	0.0%	0.0%	0.1%	0.0%
Out-of-court (formal)	1.1%	0.3%	0.2%	0.3%	1.2%
Out-of-court (informal)	1.8%	0.3%	0.3%	0.9%	2.1%
Prosecution prevented or not in the public interest	2.1%	2.1%	0.1%	0.3%	0.9%
Evidential difficulties (suspect identified; victim supports action)	17.4%	13.7%	6.6%	5.0%	7.4%
Evidential difficulties (victim does not support action)	41.5%	33.2%	20.4%	8.1%	16.8%
Investigation complete - no suspect identified	13.5%	13.2%	39.4%	71.4%	58.4%
Action undertaken by another body/agency	2.2%	2.7%	0.1%	0.1%	0.3%
Further investigation to support formal action not in the public interest	0.9%	1.2%	0.1%	0.5%	0.5%
Diversionary, educational or intervention activity, resulting from the crime report, no further action	0.7%	0.4%	0.1%	0.1%	0.3%
Not yet assigned an outcome	13.5%	30.2%	26.1%	9.0%	7.7%
Conviction ratio (%)*	71.6%	55.5%	62.9%	83.0%	67.6%
Multiplier 2	13.10	32.26	15.81	19.02	14.66

Source: Q1.3 (Flatley, 2021). Notes: * the source states that the conviction ratio is calculated using the number of crimes charged and the number of convictions in a given time period; these estimate do not necessarily relate to the same cases, as some crimes will take a long period to progress from a charge to a conviction. This suggests the data may be inaccurate, but there is no reason to believe that it will bias the data.

##### 2.7.1.3 Impacts of reoffending

Home Office estimates of the economic impact of offences are published in the Economic and Social Costs of Crime 2018 (Heeks et al., 2018). Their estimates include the impact on victim wellbeing. In using these estimates, we assumed that the economic impact of re-offences is the same as the impact of offences in general. The Economic and Social Costs of Crime 2018 report only includes crimes that relate to individual victims, it does not include ‘crimes against society’ such as motoring and drug offences (Heeks et al., 2018). These crimes have a modest economic impact and will be rarer in this cohort, restricted to those who have committed an index offence against an individual, so excluding them is likely to have a small impact.

The estimates are highly skewed. Homicide is associated with an economic cost of £3,217,740 per case and there’s a very low number of homicides each year (Table 5). Fraud and cybercrime are associated with high volumes and lower average economic costs. Estimates on the impact of restorative justice in reducing reoffending apply to reoffending as a whole; data are not available to establish whether restorative justice reduces specific reoffence types more than others. The combination of this uncertainty, and the skewed distribution of costs associated with different crime types, creates the potential for highly uncertain estimates of the economic impact of restorative justice interventions. This research therefore adopted a trimmed mean cost of reoffending, by excluding homicide, fraud and cybercrime from estimates of the mean cost of reoffending. This approach is recommended by *Pro-Bono* Economics (Pro Bono Economics, 2019).

The estimates do not include the impact of offending and sentencing on offender wellbeing. Improving the wellbeing of offenders is an important benefit of reducing offending. By excluding these benefits, the Home Office underestimates the economic and social costs of crime.

#### 2.7.2 Direct wellbeing benefits to the index victim

There is a wealth of evidence that victims say that they benefit from participating in restorative justice interventions (Shapland et al., 2007; Strang et al., 2013). They say they blame themselves less, value receiving a sincere apology and have a reduced desire for revenge (Strang et al., 2013). These benefits persist, with victims reporting statistically significant reductions in anxiety, anger and bitterness, when surveyed 10 years after participation (Sherman et al., 2015).

It was challenging to model these benefits appropriately within this economic evaluation. Two options for formally incorporating these data were explored and found not to be feasible. The first was to conduct a cost-effectiveness analysis with results presented as a ratio between costs and wellbeing benefits. The second option was to conduct a cost–benefit analysis, with wellbeing benefits transformed onto a monetary scale. An example of cost-effectiveness approach would be to present an incremental cost-effectiveness ratio (ICER) between costs and Quality-Adjusted Life Years (QALYs); an example of a cost–benefit approach would be to transform an incremental cost-effectiveness ratio onto a net benefit scale, using a cost-effectiveness threshold.

A cost-effectiveness approach would be associated with a number of challenges. It would require accounting for all victim welfare benefits on a single scale. There is no consensus on what the scale would be, and any scale chosen is likely to provide only a partial representation of the overall benefits of restorative justice for victim wellbeing. Also the survey designs are not consistent across studies, so it is challenging to synthesize data on victim wellbeing benefits. This poses a limitation for the generalization of any findings in relation to the direct benefits of restorative justice to victims. Victim welfare benefits tend to be presented on binary^1^, or ordinal^2^, scales and it is challenging to present a ratio of costs and effects using these scales. Finally, in the absence of an agreed cost-effectiveness threshold and evaluations of alternative interventions using the same scale, the results of a cost-effectiveness analysis can be difficult to interpret.

A cost–benefit approach has the advantage that multiple measures of victim wellbeing could potentially be included in the same analysis. Whilst that would circumvent one of the challenges associated with the cost-effectiveness approach, all of the other challenges outlined would also apply. There would also be additional challenges associated with assigning a financial value to victim wellbeing benefits. There is not a universally agreed scale for measuring the effectiveness of victims’ services. Estimating a cost-effectiveness threshold for restorative justice would require calculating the marginal cost of victims’ services in meeting the needs of victims (Lomas et al., 2019) or a universal measure of the government’s willingness to pay for victims’ outcomes. We did not find any studies that transformed the direct benefits of restorative justice for victims onto a monetary scale. Tackling these substantial methodological issues is beyond the scope of this research.

Angel et al. (2014) measure the impact of restorative justice interventions on Post Traumatic Stress Symptoms (PTSS). The study used data from the Shapland report (Shapland et al., 2007). This could potentially be converted onto a monetary scale indirectly, using two possible methods. One option would be to map the PTSS (measured using Impact of Events (Revised) Scale (IESI)) onto a health-related quality of life (HRQoL) scale. This would have the benefit that a cost-effectiveness threshold has already been established for HRQoL measures (McCabe et al., 2008). We were not able to find any suitable mapping algorithms to achieve this. The second option would be to benchmark the benefits of restorative justice interventions in tackling PTSS, against another intervention, such as Cognitive Behavioral Therapy (CBT). Again, we were not able to find any comparisons of the effectiveness of restorative justice interventions and CBT, or any studies that measure the benefits of CBT for individuals with PTSS, using the IESI scale. Also, the impact of restorative justice on PTSS, only represents a subset of the overall benefits of restorative justice to victim wellbeing, so these methods would substantially understand the benefits of restorative justice for victims.

Instead, a pragmatic method was used to include direct benefits to the index victim within an exploratory scenario. The Home Office Economic and Social Costs of Crime report includes estimates of the impact of crime on victims’ wellbeing (Heeks et al., 2018). It will be rare for restorative justice interventions to leave victims better off than they would have been, had they not become a victim of crime. This is in part because restorative justice takes place after the crime, so some of the harm has already been inflicted. We conducted a scenario analysis where restorative justice participation was assumed to reduce the total harm experienced as a result of being a victim of crime by an assumed fraction.

Restorative justice service managers advised that victims can benefit from receiving support at the referral or assessment stage, even if they do not progress to participating in a restorative justice intervention. This is analogous to a victim receiving support from a victim support service. We modeled a scenario in which the cost of the assessment stage was removed. This is equivalent to assuming that victims completing the referral and assessment phases would otherwise need to receive support from victim services, which have an equivalent cost.

#### 2.7.3 Direct wellbeing benefits to the index offender

Whilst there is substantial evidence that participating in restorative justice interventions directly benefits offender wellbeing (Sherman et al., 2015), it was not of a form that could be incorporated within this research. Data were not available in a format that could be incorporated within an economic model and the appropriate method to do so was unclear.

All of the challenges outlined in Section 2.6.2 also apply here and there are additional challenges too. Evidence suggests that offenders benefit from participating in restorative justice interventions but how this benefit should be measured is complex and contentious. One of the stated aims of these interventions is to allow offenders to share in the emotional impacts victims experience and to feel shame for their actions (Sherman and Strang, 2007). To try to measure the impact of a restorative justice intervention by asking an offender whether their wellbeing has increased may be an inappropriate way of synthesizing the complex emotional impact of offender participation. How best to account for the direct impact of restorative justice on offenders is an important area for future research.

### 2.8 Costs

There are a number of different possible methods for estimating the costs associated with restorative justice. Resource constraints and a lack of data made it challenging to estimate the cost of delivering restorative justice robustly. The different methods explored and the justification for the chosen method are presented in the Supplementary material. Multiple restorative justice services provided resource-use estimates for the different stages in the restorative justice pathway (Supplementary material). The estimates used in the base case are presented in Table 6. An exploratory scenario was implemented in which the overall cost of delivering restorative justice was increased by 100% to account for the cost of conducting restorative justice training and raising awareness of restorative justice.

**Table 6:** Cost estimates for the restorative justice pathway.

Stage in the process	Restorative justice worker time	Restorative justice manager time	Police constable	Total cost
Referral	45 min	9 min	–	£15
Assessment	7 h	1.4 h	15 min	£158
Direct restorative justice	79 h	15.8 h	–	£1,592
Indirect restorative justice	19 h	3.8 h	–	£373

### 2.9 Analysis

The primary results were presented as a social benefit–cost ratio of investment in restorative justice. The costs and benefits were broken down by stakeholder (criminal justice system, government, society). An analysis was conducted for a cohort of cases, presenting the number of restorative justice interventions delivered, and of each offence type avoided, when a defined cohort was referred to a restorative justice service.

**Figure 3:**
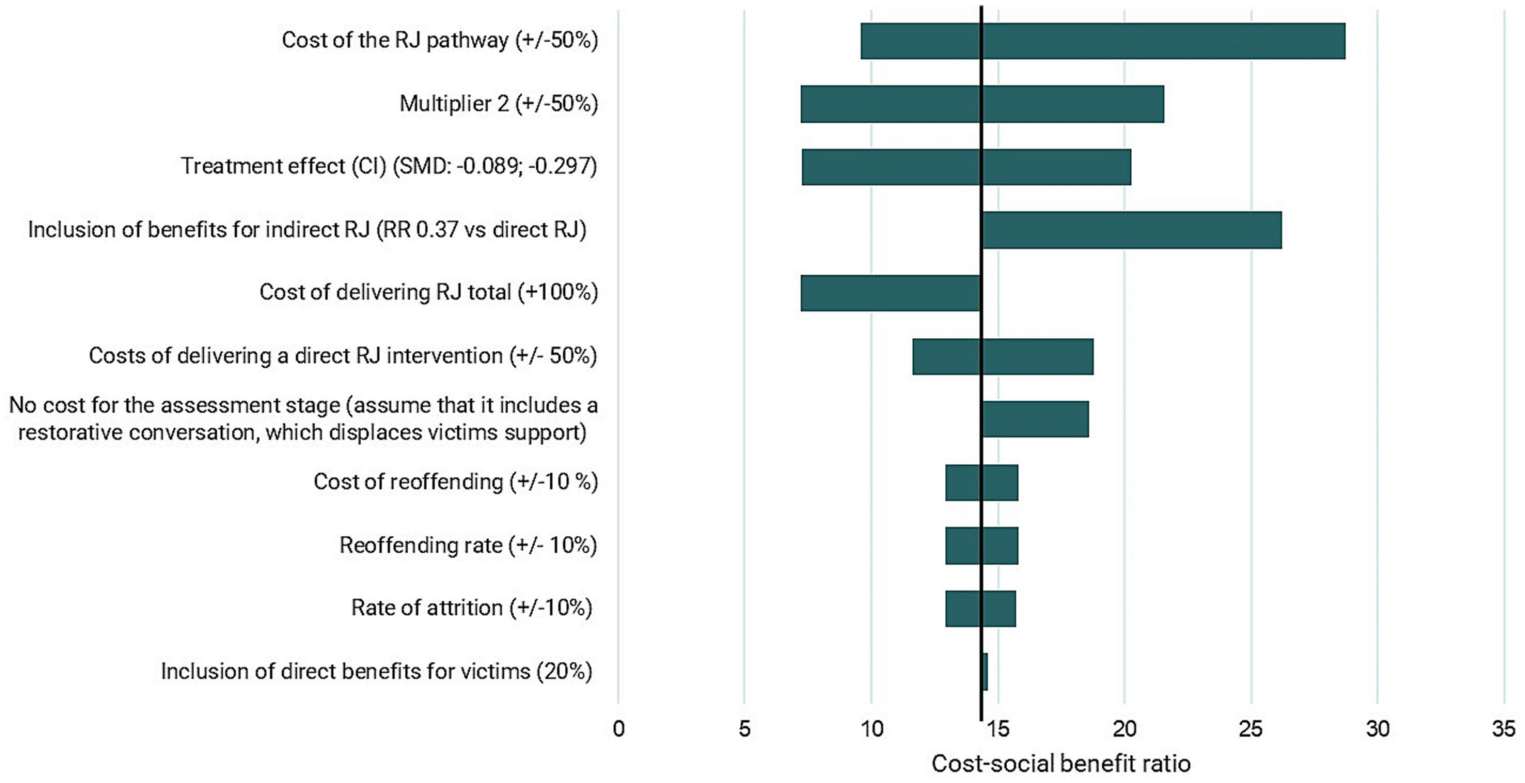
Tornado diagram displaying sensitivity and scenario analysis (£). Source: Grimsey Jones and Harris (2022).

A sensitivity analysis was conducted to test the sensitivity of the model results to changes in the inputs and assumptions. Results are presented in Figure 3. Sensitivity analyses were run regarding the economic impact of reoffending, the baseline reoffending rate and the relationship between proven reoffending and reported reoffending. The cost of delivering restorative justice and the rate of attrition in the restorative justice pathway were also both varied in the sensitivity analysis. Scenarios were run in which direct benefits to the index victim were included, indirect restorative justice was assumed to be associated with reductions in reoffending (as well as direct), and the persistence of reductions in reoffending was increased. A subgroup analysis was conducted for different index offence types.

## 3 Results

### 3.1 Base case

The modeled social benefit–cost ratio in the base case was £14 per £1 spent (Table 7). The financial return on investment for the criminal justice system was £4 per £1 spent. The cost of delivering a direct restorative justice intervention was £3,394. It cost £420 per crime avoided, with eight crimes avoided per direct restorative justice intervention and 0.68 offences avoided per referral.

**Table 7:** Base case estimates of the social benefit–cost ratio of restorative justice.

	Total cost per referral	Total cost per direct restorative justice intervention	Total benefit per referral	Total benefit per restorative justice intervention	Benefit–cost ratio (societal)	Benefit–cost ratio (Criminal justice system)
Base case	£284.73	£3,394.25	£4,092.42	£48,785.53	£14.37	£4.23

**Figure 4:**
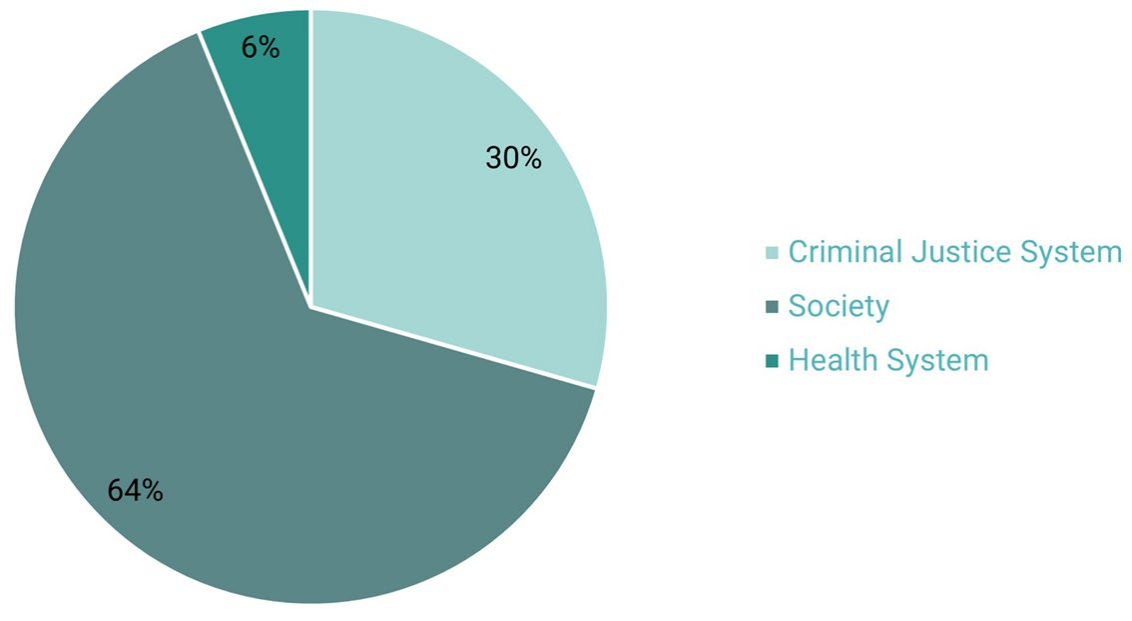
Total benefits by stakeholder. Source: Grimsey Jones and Harris (2022).

The criminal justice system received 30% of the benefits, in the form of financial savings (Figure 4). The majority of the benefits accrued to society in the form of economic and social benefits of reduced crime.

### 3.2 Cohort of 100 cases referred results

Results were modeled for a typical cohort of 100 cases referred to a restorative justice service (Table 8). In the model, 10 direct restorative justice interventions took place, resulting in 74 reoffences being avoided. There was a modeled total social benefit of £409,242 and a net social benefit of £380,957 when the costs of delivering restorative justice were accounted for.

**Table 8:** Results for a cohort of 100 cases referred.

Offence type	No. of cases referred	No. of direct interventions delivered	Total cost	No. of total reoffences avoided	Total benefit
Violence against the person	50.73	4.26	£14,444	20.9	£169,276
Sexual	2.19	0.18	£624	0.63	£4,846
Robbery	1.94	0.16	£553	0.96	£7,828
Theft	20.52	1.72	£5,843	32.00	£153,331
Criminal damage and arson	24.61	2.06	£7,007	13.28	£73,916
Total	100.00	8.39	£28,473	74.25	£409,242

### 3.3 Sensitivity analysis

The results of the sensitivity analysis are presented in Table 9 and Figure 3. The analysis associated with the largest change in the results was where the costs of delivering the restorative justice pathway were increased, or decreased, by 50%. This resulted in a social benefit–cost- ratio £9.58 per £1 when the costs were reduced, and £28.75 per £1 when the costs were increased. Where Multiplier 2, the relationship between police-reported crime and total crime, was increased (decreased) by 50% this was associated with a social benefit–cost- ratio of £7.19 (£21.56) per £1. Varying the treatment effect, the reduction in reoffending associated with completing a restorative justice intervention, was associated with a social benefit–cost ratio of £7.25 per £1 with the treatment effect at its lower limit and £20.27 per £1 with the treatment effect at its upper limit.

**Table 9:** Sensitivity analysis.

No.	Analysis	Lower estimate	Base case	Upper estimate
1	Cost of the RJ pathway (+/−50%)	£9.58	£14.37	£28.75
2	Multiplier 2 (+/−50%)	£7.19	£14.37	£21.56
3	Treatment effect (CI) (SMD: −0.297; −0.089)	£7.25	£14.37	£20.27
4	Inclusion of benefits for indirect RJ (RR 0.37 vs. direct RJ)	–	£14.37	£26.21
5	Cost of delivering RJ total (+100%)	£7.19	£14.37	–
6	Costs of delivering a direct RJ intervention (+/− 50%)	£11.64	£14.37	£18.78
7	No cost for the assessment stage (assume that it includes a restorative conversation, which displaces victims support)	–	£14.37	£18.59
8	Cost of reoffending (+/−10%)	£12.94	£14.37	£15.81
9	Reoffending rate (+/− 10%)	£12.94	£14.37	£15.81
10	Rate of attrition (+/−10%)	£12.91	£14.37	£15.72
11	Inclusion of direct benefits for victims (20%)	–	£14.37	£14.58
A1	Reduction in reoffending for 2 years	–	£14.37	£29.02
A2	Reduction in reoffending for 3 years	–	£14.37	£42.88
A3	Reduction in reoffending for 4 years	–	£14.37	£57.27

Assuming the effectiveness of indirect restorative justice was 37% of the effectiveness of direct restorative justice (instead of no effect), increased the social benefit–cost ratio to £26 per £1. Doubling the costs of delivering restorative justice, to account for the fixed costs of managing a restorative justice service, yielded a social benefit–cost ratio of £7.19. When the cost of the assessment stage was removed, to model an assumption that victims would otherwise have received support from victim services, the social benefit–cost ratio increased to £18.59 per £1. When the cost of reoffending, or the reoffending rate, were increased (decreased) by 10%, this yielded a social benefit–cost ratio of £12.94 (£15.81) per £1. When the rate of attrition was increased (decreased) by 10% this was associated with a social benefit–cost ratio of £12.91 (£15.72) per £1. Finally, including direct benefits to the index victim, increased the social benefit–cost ratio from £14.37 to £14.58 per £1.

An exploratory scenario was conducted in which restorative justice continued to reduce reoffending over a four-year period. The results are presented in Table 9. When restorative justice was assumed to reduce reoffending over a longer time period, this was associated with substantial benefits, with an upper estimate of £57.27 per £1 when the reduction in reoffending was assumed to persist for 4 years.

## 4 Discussion

This research presents the first economic model for restorative justice, involving adults or youths, who have committed proven offences in England and Wales. England and Wales were the focus due to regional differences in the criminal justice systems across the UK. The research substantially advances understanding of the economic impact of restorative justice, particularly the key drivers of the economic impact. It establishes a framework for conducting economic evaluations of restorative justice, and other preventative criminal justice interventions. The research strengthens the economic case for investment in restorative justice, suggesting there could be important economic benefits for a range of stakeholders. The modeled social benefit–cost ratio in the base case of £14 per £1 invested, is substantial. The evidence underpinning these results is strong relative to other criminal justice interventions. Of particular importance is that the impact of restorative justice on reoffending is taken from a meta-analysis of an international program of randomized control trials (Strang et al., 2013).

The estimated base case social benefit–cost ratio in this study exceeded the estimates in the Shapland report of £8 per £1 invested, for the JRC schemes (Shapland et al., 2008). One contributing factor is that the scope of the Home Office estimates for the social and economic costs of crime, also used within the Shapland report, has been expanded to include victim wellbeing (Heeks et al., 2018). This yielded increased estimates of the economic benefit of reducing crime. A further contributor is that the Shapland report offence mix included within the Shapland report was aligned with that observed within the trials. This study included cases with a mix of index offence types that match the mix of proven offences in England and Wales (Flatley, 2021). The cost per intervention was at the lower end of the range of estimates in the Shapland report, (£3,372 compared with £2,984 to £5,963 [inflated from 2005 to 2021 costs]) (Shapland et al., 2008). The estimated financial savings were somewhat higher (£53,485 compared with £44,969 [inflated from 2005 to 2021 costs]) (Shapland et al., 2008). This approximate alignment provides external validation for the results of this research.

Several areas of uncertainty were tested in a sensitivity analysis. Some of these sensitivity analyses demonstrated a linear 1:1 relationship between the input being varied and the outputs of the analysis. Where the reoffending rate, cost of reoffending and Multiplier 2 were varied in sensitivity analysis (No. 2, 3, and 10) increasing (or decreasing) the input yielded a proportionate increase (or decrease) in the results. This means that the sensitivity of the results to these variables is high, and as some of these inputs are highly uncertain, the base case results are also highly uncertain. The cost of delivering restorative justice, from referral to intervention, was the most important source of uncertainty. We varied these costs by ±50%, as data and expert opinion suggested the costs of delivering restorative justice interventions are varied and uncertain. One of the challenges was that a large number of stakeholders can contribute to the restorative justice pathway and there is a lack of data on resource use by these stakeholders. A further challenge was that restorative justice services conduct a range of work, including training and raising awareness of restorative justice, that is not linked to individual restorative justice cases. This was challenging to account for within an economic model constructed at the individual case level. Nevertheless, these activities are an integral part of running a restorative justice service and are thus one component of the cost of delivering restorative justice. The social benefit–cost ratio of restorative justice remained substantial (£11 per £1), even when the cost of delivering restorative justice was increased by 50%, suggesting results were robust to this uncertainty. Further research on the costs of delivering restorative justice interventions would help to reduce this uncertainty.

Whether, and to what extent, indirect restorative justice interventions reduce reoffending was an important driver of the cost-social benefit ratio. All restorative justice services that were consulted, deliver a higher proportion of indirect restorative justice interventions than direct restorative justice interventions. Direct restorative justice interventions are often not possible, due to the preference of the victim, the preference of the offender or logistical challenges. The Shapland report did not find a statistically significant relationship between indirect restorative justice interventions and reoffending. The Strang et al. (2013) meta-analysis did not include cases of indirect restorative justice. There are reasons to believe these interventions are less effective, including that in the Shapland trials 66% of victims and 91% of offenders said it was important to speak directly (Shapland et al., 2006).

The inclusion of a hypothetical assumed direct benefit for victims as a result of participating in a restorative justice intervention resulted in a surprisingly small increase in the social benefit–cost ratio. This scenario utilized estimates of the impacts of different offence types on victim wellbeing from the Economic and Social costs of Crime report (Heeks et al., 2018). There are a number of reasons that this method could underestimate the benefits of restorative justice participation for victims. Restorative justice services support victims of proven crimes, not all crimes, these crimes may have a higher than average impact on victim wellbeing. Also, only some victims actively pursue restorative justice participation and these victims may have experienced greater than average harm as a result of being victims. Finally, it is possible that the offender has committed multiple crimes against a single victim. For all these reasons, the estimated harm experienced by victims participating in restorative justice may be underestimated, meaning that the potential direct benefit of restorative justice to the victim, could be underestimated. Whilst there is substantial data showing that restorative justice interventions benefit victims, including over the long term, it is not of a form that can feasibly be incorporated within an economic evaluation (Sherman et al., 2015). There is a need for improved data on the direct benefits of restorative justice for victims, using methods that support economic evaluation of these benefits (Heeks et al., 2018).

How long restorative justice interventions reduce reoffending for was an important area of uncertainty. In the base case it was assumed that reoffending was only reduced for 1 year, to align with the length of the restorative justice trials and baseline reoffending data. It is likely that the reduction in reoffending for offenders engaging in restorative justice would continue for some time after the length of the trials; it would gradually converge on the reoffending rate of those not engaging in restorative justice over time (Sherman et al., 2015). Greater evidence is needed on the long term relationship between restorative justice participation and reoffending.

### 4.1 Limitations

There are a number of limitations associated with the methodology of this economic evaluation. These are a result of the limitations of the evidence base, complexity of the subject matter and the resource limitations of the research project.

The most important limitation is that this research synthesizes a range of different sources of data and evidence. There is a high degree of misalignment between the scope of these sources, meaning a substantial risk of bias. This is unavoidable, given the nature of the available evidence. For example, the mix of index offence types differs between sources (Strang et al., 2013; Heeks et al., 2018). In the majority of cases, experts have indicated that the risk of bias in these instances is modest. Further research on the determinants of the benefits of restorative justice, would improve the validity of this analysis.

Some parameters within the economic model were informed by data provided by restorative justice services, including police forces and independent providers. This includes the rates of attrition and the rates of resource use at the different stages of the restorative justice pathway. Estimated rates of attrition were informed by small numbers of cases and there were challenges aligning data collected by restorative justice services with the inclusion criteria of this study. Resource use was predominately informed by managers of restorative justice services, which is likely to be subject to optimism bias.

There are a number of gaps in the evidence on the benefits of restorative justice. The best available evidence on the impact of restorative justice interventions on reoffending is relatively old (Shapland et al., 2008; Strang et al., 2013). Most of the evidence from high-quality sources is not of a form that can be incorporated within an economic evaluation and is relatively short-term (Shapland et al., 2008; Strang et al., 2013).

The relationship between proven reoffences and total reoffences is highly uncertain. If proven reoffences represent a small fraction of total reoffences, and restorative justice reduces both equally, the return on investment of restorative justice interventions increases. It is unlikely that additional research would be able to substantially reduce uncertainty in this area, because the majority of total reoffences are almost by definition unattributable. It is possible that trial data on reductions in proven reoffences could be combined with offender self reports as to their total reoffending, including unproven reoffending.

## 5 Conclusion

This research suggests that restorative justice has the potential to yield a substantial social benefit–cost ratio and direct return on investment to the criminal justice system. Whilst the existing evidence is strong, there are a number of areas in which additional research is important. This would advance policymakers’ understanding of the value of restorative justice as well as how to harness this value to benefit victims, offenders and society. A number of policy changes are needed to improve collective understanding. This includes improved national data collection on restorative justice provision, national guidance on the definition of key terms (e.g., referral, case and conference) and standardized and mandatory evaluation of services.

Future research should focus on several key areas. This includes improved and standardized measurement of the wellbeing of victims and offenders, including the impact of restorative justice on wellbeing, understanding the impact of the diversity of restorative justice interventions delivered within England and Wales, and more accurate bottom-up costing of restorative justice interventions. There is a need for improved data collection across the sector: greater national collation and standardization of data, and better-quality data. This will help to facilitate future research.

## Data availability statement

The datasets presented in this study can be found in online repositories. The names of the repository/repositories and accession number(s) can be found at: https://why-me.org/our-work/our-projects/economic-evaluation-of-restorative-justice/#:~:text=of%20Restorative%20Justice-,Why%20me%3F,with%20the%20conventional%20justice%20system.

## Author contributions

FGJ led this research project, developed the economic model, and authored the research. LH conducted the literature reviews which informed the inputs and assumptions of the economic model. LJ, JF, LA, and JS provided strategic insight regarding the approach, assumptions and presentation of the results of the research. All authors contributed to the article and approved the submitted version.

## Footnotes

^1^e.g. Percentage of victims who received a letter of apology (Shapland et al., 2007).

^2^e.g., “Thinking about what happened at the conference itself, would you say you were: very satisfied, Quite satisfied…” (Strang et al., 2013).
