# Supplementary material for: An economic evaluation of restorative justice post-sentence in England and Wales
Source: Front Psychol. 2023 Nov 16;14:1162286. doi: 10.3389/fpsyg.2023.1162286 (PMC10693425; doi:10.3389/fpsyg.2023.1162286)
Supplement: Supplementary file 1 [file Data_Sheet_1.docx]

Supplementary Material

# Evidence review

Table 1 presents a summary of the studies reviewed to source cost and resource use data and understand the methods used by previous studies to estimate these data. Table 2 presents a summary of the studies reviewed to understand the methods used by past economic evaluations of restorative justice.

**Table 1: Cost and resource use of restorative justice review summary table**

| **Study** | **Summary** |
| --- | --- |
| Birmingham Scheme Evaluation (Macdonald et al., 2017). | **Population:** 287 cases; anti-social behaviour and neighbourhood disputes cases amongst social housing tenants.  **Intervention:** face-to-face Restorative Justice conference; referral to ‘the Restorative Justice specialist team’ then housing officers, PCs or PCSOs to manage conference.  **Comparator:** case closed/ civil intervention for ASB; comparison with case records relating to ‘standard’ anti-social behaviour of same time period; dataset includes 897 cases from 2010-16.  **Outcomes:** reduction in repeat disputes in 83% of cases (of only 74 cases for which data was collected); in 93% of cases both parties satisfied after conference; quicker process with comparator cases taking 117 days vs 27 for Restorative Justice cases.  **Study Design:** Birmingham Social Housing Partnership managed Restorative Justice project; Phase 1 compared costs of Restorative Justice conferences for agencies involved. Phase 2 explored the experiences of participants; control group constructed from previous data.  **Cost and resource use:** Lowest complexity cases: £240 per case (£268 2021 pounds). Median estimate: £382 per case (£427 2021 pounds). Highest estimate: £515 per case (£574 2021 pounds). |
| Greater Manchester Police, Restorative Justice Business Case, (Shewan, 2010) | **Population:** young offenders (under 18) with low-level antisocial and ‘nuisance’ offences; cases processed in a range of UK police forces.  **Intervention:** diversion from standard youth justice procedures including reprimand and custody to RJ conferencing.  **Comparator:** youth justice standard processes including reprimand and custody.  **Outcomes:** produced cost analysis of RJ conferencing schemes, showing net cost savings through reductions in police staff time.  **Study Design:** evaluation of RJ including reoffending, victim satisfaction, value for money and public confidence; data taken from 33 police forces and 8 forces in the ‘Youth Restorative Disposal’ pilot scheme.  **Cost and resource use:** Estimates a cost of £15.95 and £20.21 for a ‘Level 1’ RJ (minor offence). RJ conference cost of £87.15 and £5,803. |
| Institute for Criminal Policy Research Evaluation (Kirby & Jacobson, 2015) | **Population:** 55 pre-sentence RJ conferences and 38 alternative RJ activities; serious acquisitive and violent cases.  **Intervention:** additional not diversionary; pre-sentence restorative justice to victims and offenders in ten Crown Courts in England and Wales.  **Comparator:** standard Crown Court processes.  **Outcomes**: evaluation of the scheme, including numbers of cases processed per site, rates of attrition, and qualitative data on victim and offender experiences.  **Study Design:** evaluation of a pre-sentence restorative justice pathfinder programme.  **Cost and resource use:** Average of 110 hours per case of project manager time, plus 6 hours per case from police, prison service, Crown Prosecution Service and court staff combined. These are underestimates as additional supervision time of the project manager, and volunteer time, are referred to but not reported. |
| Matrix Evidence Report (Matrix Evidence, 2009) | **Population:** young adult offenders (18-24) sentenced in a Magistrate’s court for a non-violent offence.  **Intervention:** diversion from community orders to pre-court RJ conferencing schemes.  **Comparator:** community sentences given at Magistrate’s Court.  **Outcomes:** produced cost analysis of RJ triage scheme; schemes analysed produced net cost savings to society through public sector costs avoided and reduced victim costs.  **Study Design:** economic analysis of alternative interventions for young adult offenders, including RJ schemes; costs analysed using secondary data.  **Cost and resource use:** Total RJ conferencing scheme cost per offender: £850 (£1,088 2021 pounds). |
| Newcastle Scheme Evaluation (Soppitt & Irving, 2011) | **Population:** 190 young offenders (age 10-17); Newcastle Youth Offending Team; offences include theft and handling stolen goods; public order; criminal damage; and violence against the person.  **Intervention:** ‘Triage’ diversion of first-time entrants into CJ, including into RJ procedures.  **Comparator:** standard criminal justice procedures including reprimand and final warnings in Youth Offending Teams.  **Outcomes:** estimated a cost-social benefit ratio linked to changes in reoffending rates.  **Study Design:** economic analysis of RJ triage scheme based on reoffending ratio between RJ and CJ; Newcastle upon Tyne.  **Cost and resource use:** Not explicitly reported. |
| Study: Shapland 2008 (three schemes reported separately) (Shapland et al., 2008) | |
| Scheme: Connect | **Population:** 50 cases of completed RJ; adult offenders from magistrates’ courts cases between conviction and sentence, some referrals from victims and Crown court cases; offences range from assaults (34%), burglary (24%), theft/taking a vehicle (10%), and criminal damage (8%).  **Intervention:** indirect mediation/ direct mediation/ conferencing; additional not diversionary.  **Comparator:** matched characteristics control group from Magistrates’ Court cases.  **Outcomes:** control group reconvicted more (not significant); no significant frequency of reconviction; RJ group higher severity of reconviction but not significant; no significant reconviction in terms of costs but direct control more cost savings and indirect RJ group more cost savings.  **Study Design:** Inner London; individual matching process to generate control group using Magistrates’ Court records.  **Cost and resource use:** Start-up phase - cost per case referred: £4,447, cost per case with RJ completed: £9,530 (£15,866 2021 pounds). Running phase - cost per case referred: £1,360, cost per case with RJ completed: £4,351 (£6,667 2021 pounds). Direct mediation average £8,739 per case and indirect mediation £2,832 per case. |
| Scheme: REMEDI | **Population:** 132 cases; adult cases community sentences during resettlement and prison pre-release and during long sentence with most offence types violence (22%), burglary (20%) and theft and handling (19%); youth final warning cases, referral orders and youth justice sentences with most offence types theft and handling (29%), violence (23%) and criminal damage (22%), burglaries (11%).  **Intervention:** indirect mediation and direct mediation; additional not diversionary.  **Comparator:** matched characteristics control group using previous records.  **Outcomes:** no significant reconviction rates (but adult indirect and youth direct RJ reconvicted more); no significant frequency of reconviction; RJ group higher severity of reconviction for adults and control group for youth but neither significant; no significant difference in reconviction in terms of cost but control more cost savings except in youth indirect.  **Study Design:** South Yorkshire (Sheffield and Doncaster); Matched control group to each individual offender in the restorative justice group.  **Cost and resource use:** Start-up phase (Adult/Youth) - cost per case referred: £596/£312, cost per RJ completed case: £9,143/£1,726. Running phase (Adult/Youth) - cost per case referred: £221/£324, cost per completed RJ case: £2,908/£1,944 (£4,666/ £3,088 2021 pounds). |
| Scheme: JRC London | **Population:** 106 adult attempted/ robbery and theft cases and 186 adult burglary of a dwelling cases; cases being tried at Crown court.  **Intervention:** RJ conferences; post-guilty plea and pre-sentence; additional not diversionary.  **Comparator:** no RJC pre-sentence; tried at Crown Court.  **Outcomes:** significantly greater cost reduction in terms of reconvictions for burglary cases but not for robbery cases; no significant differences between intervention and control group for rates of recidivism, severity of reconviction, and frequency of reconviction.  **Study Design:** random control trials with cases randomly allocated to either conference or control group; two-year follow-up period.  **Cost and resource use:** Original estimate Start-up phase: Cost per case referred: £1,902, cost per completed RJ case: £5,949 (£9,896 2021 pounds). Running phase: Cost per case referred: £1,221, cost per randomly assigned case: £3,797. |
| Scheme: JCR Thames Valley | **Population:** 114 adult offenders in prison and within 12 months of release, and 65 adult offenders given a community sentence at the magistrates’ court; violent offence types including common assault and ABH.  **Intervention:** RJ conferences in prison post-release and pre- and post-sentence period on probation for the community sentences; additional not diversionary.  **Comparator:** no RJC in prison or on probation; community sentence at Magistrates Court  **Outcomes:** statistically significant reductions in number of offences committed for both intervention and control groups for prison population; no significant differences between intervention and control group for costs of reconviction, rates of recidivism, severity of reconviction, and frequency of reconviction.  **Study Design:** randomised control trials with cases randomly allocated to either conference or control group; two-year follow-up period.  **Cost and resource use:** Start-up costs - cost per case referred: £952, cost per case RJ completed: £6,188(£10,184, 2021 pounds). Running phase - cost per case referred: £333, cost per randomly assigned case: £2,831. |
| Scheme: JRC Northumbria | **Population:** 206 youth offenders given final warning and 107 adult cases from Magistrates Court; property and violent offences.  **Intervention:** diversion to a police caution with RJC for youth; post guilty plea and pre-sentence for adults. Comparator: diversionary proposal to caution with no RJC for youth; no RJC pre-sentence and sentencing in Magistrates Court for adults.  **Outcomes:** statistically significant reductions in recidivism and severity of reconviction for adult property offenders; no significant reductions in frequency of reconviction or reconvictions in terms of cost.  **Study Design:** random control trials with cases randomly allocated to either conference or control group; control group was established during the running of the scheme; two year follow-up period.  **Cost and resource use:** Start-up phase - cost per case referred: £1,467, cost per RJ completed case £5,755 (£9,555 2021 pounds). Running phase - cost per case referred: £557, cost per randomly assigned case: £1,898. |
| Surrey Scheme Evaluation (Mackie et al., 2014) | **Population**: young offenders under 18 (35% under 16); 25% male offenders; not serious offences with theft and handling, and violence most common; control group constructed from pre-court and court convictions 2009-11.  **Intervention:** Youth Restorative Intervention; conferencing, direct or indirect mediation; pre-court disposal.  **Comparator:** alternative to youth caution, youth conditional caution or prosecution (respectively).  **Outcomes:** 91% victim satisfaction with involvement; reduction in first time entrants (from 394 in 2010-11 to 189 in 2012-13); lower reoffending rates in intervention group (27%) than control group (33%) with 18% reduction.  **Study Design:** YRI run by Surrey Police and Surrey County Council’s Youth Support Service; default assignment to YRI, control group constructed from historic data  **Cost and resource use:** cost to administer per offender: £360 (£418 2021 pounds). |
| US Scheme, Economic Evaluation (Furman, 2012) | **Population:** adult offenders (196 RJ and 1,786 conventional justice (CJ) cases) in Massachusetts, including property crimes, substance-related charges, theft/attempted theft charges, and assault/crimes against persons.  **Intervention:** alternative and diversion RJ procedures, sample from Communities for Restorative Justice 2000-9 data.  **Comparator:** ‘traditional’ criminal justice procedures, sample from 2008 Males Release Cohort data.  **Outcomes:** produced cost analysis of RJ, showing net cost savings through reductions in reoffending.  Cost-social benefit ratio of $3.13 per $1 spent.  **Study Design:** cost-effectiveness analysis/economic evaluation of RJ using recidivism rates as ‘effectiveness’ variable; Massachusetts, US.  **Cost and resource use:** |

**Abbreviations:** CJ, conventional justice ; RJ, restorative justice; RJC, Restorative Justice Congress

**Table 2: Economic evaluation of restorative justice review summary table**

| **Study** | **Summary** |
| --- | --- |
| Birmingham Scheme Evaluation (Macdonald et al., 2017) | **Population:** 287 cases; anti-social behaviour and neighbourhood disputes cases amongst social housing tenants.  **Intervention:** face-to-face RJ conference; referral to ‘the RJ specialist team’ then housing officers, police constables or police community support officers to manage the RJ conference.  **Comparator:** case closed/ civil intervention for anti-social behaviour; comparison with case records relating to ‘standard’ anti-social behaviour of same time period; dataset includes 897 cases from 2010-16.  **Outcomes:** reduction in repeat disputes in 83% of cases (of only 74 cases for which data was collected); in 93% of cases both parties were satisfied after the conference; quicker process with comparator cases taking 117 days vs 27 for RJ cases.  **Study Design:** Birmingham Social Housing Partnership managed RJ project; Phase 1 compared costs of RJ conferences for agencies involved. Phase 2 explored the experiences of participants; control group constructed from previous data.  **Results:** Mean cost-social benefit ratio of £9.95 for every £1 spent. With a range from £21.60 to £4.95. High complexity cases - £21.60 Medium complexity cases - £4.95 Low complexity cases - £7.75 |
| Greater Manchester, Restorative Justice Business Case (Shewan, 2010) | **Population:** young offenders (under 18) with low-level antisocial and ‘nuisance’ offences; cases processed in a range of UK police forces.  **Intervention:** diversion from standard youth justice procedures including reprimand and custody to RJ conferencing.  **Comparator:** youth justice standard processes including reprimand and custody.  **Outcomes:** produced cost analysis of RJ conferencing schemes, showing net cost savings through reductions in police staff time.  **Study Design:** evaluation of RJ including reoffending, victim satisfaction, value for money and public confidence; data taken from 33 police forces and 8 forces in the ‘Youth Restorative Disposal’ pilot scheme.  **Results:** Cost-social benefit ratio £7.18 for every £1 spent. Hertfordshire: £8.39. Cheshire: £7.77. North Wales: £5.37. |
| Matrix Evidence Report (Matrix Evidence, 2009) | **Population:** young adult offenders (18-24) sentenced in a Magistrate’s court for a non-violent offence.  **Intervention:** diversion from community orders to pre-court RJ conferencing schemes.  **Comparator:** community sentences given at Magistrate’s Court.  **Outcomes:** produced cost analysis of RJ triage scheme; schemes analysed produced net cost savings to society through public sector costs avoided and reduced victim costs.  **Study Design:** economic analysis of alternative interventions for young adult offenders, including RJ schemes; costs analysed using secondary data.  **Results:** Cost-social benefit ratio of £10.71 for every £1 spent. |
| Newcastle Scheme Evaluation (Soppitt & Irving, 2011) | **Population:** 190 young offenders (age 10-17); Newcastle Youth Offending Team; offences include theft and handling stolen goods; public order; criminal damage; and violence against the person.  **Intervention:** ‘Triage’ diversion of first-time entrants into CJ, including into RJ procedures.  **Comparator:** standard criminal justice procedures including reprimand and final warnings in Youth Offender Teams..  **Outcomes:** conducted a cost-social benefit ratio analysis and estimate, showing cost savings of future offences based on reoffending rates.  **Study Design:** economic analysis of RJ triage scheme based on reoffending ratio between RJ and CJ; Newcastle upon Tyne.  **Results:** Cost-social benefit ratio of £2.30 per £1 spent. |
| Study: Shapland 2008 (three schemes reported separately) (Shapland et al., 2008) | |
| Scheme: CONNECT | **Population:** 50 cases of completed RJ; adult offenders from Magistrates’ Court cases between conviction and sentence, some referrals from victims and Crown Court cases; offences included assaults (34%), burglary (24%), theft/taking a vehicle (10%), and criminal damage (8%).  **Intervention:** indirect mediation/ direct mediation/ conferencing; additional not diversionary.  **Comparator:** matched characteristics control group from Magistrates’ Court cases.  **Outcomes:** control group reconvicted more (not significant); no significant frequency of reconviction; RJ group higher severity of reconviction but not significant; no significant reconviction in terms of costs but direct control more cost savings and indirect RJ group more cost savings.  **Study Design:** inner London; individual matching process to generate control group using Magistrates’ Court records.  **Results:** Negative cost-social benefit ratio of £2.23 for every £1 spent. |
| Scheme: REMEDI | **Population:** 132 cases; adult cases community sentences during resettlement and prison pre-release and during long sentence with the following offence types: violence (22%), burglary (20%) and theft and handling (19%); youth final warning cases, referral orders and youth justice sentences with predominantly the following offence types: theft and handling (29%), violence (23%) and criminal damage (22%), burglaries (11%).  **Intervention:** indirect mediation and direct mediation; additional not diversionary.  **Comparator:** matched characteristics control group using previous records.  **Outcomes:** no significant change to reconviction rates (but adult indirect and youth direct RJ reconvicted more); no significant change to the frequency of reconviction; RJ group higher severity of reconviction for adults and control group for youth but neither significant; no significant difference in reconviction in terms of cost but control lower cost except in youth indirect.  **Study Design:** South Yorkshire (Sheffield and Doncaster); Matched control group to each individual offender in the restorative justice group.  **Results:** Negative cost-social benefit ratio of £8.32 for every £1 spent |
| Scheme: JRC London | **Population:** 106 adult attempted/ robbery and theft cases and 186 adult burglary of a dwelling cases; cases being tried at Crown court.  **Intervention:** RJ conferences; post-guilty plea and pre-sentence; additional not diversionary.  **Comparator:** no RJC pre-sentence; tried at Crown Court.  **Outcomes:** significantly greater cost reduction in terms of reconvictions for burglary cases but not for robbery cases; no significant differences between intervention and control group for rates of recidivism, severity of reconviction, and frequency of reconviction.  **Study Design:** random control trials - cases randomly allocated to either conference or control group; two year follow-up period.  **Results:** Cost-social benefit ratio £9.75 for every £1 spent. |
| Scheme: JRC Thames Valley | **Population:** 114 adult offenders in prison and within 12 months of release, and 65 adult offenders given a community sentence at the magistrates’ court; violent offence types including common assault and ABH.  **Intervention:** RJ conferences in prison post-release and pre- and post-sentence period on probation for the community sentences; additional not diversionary.  **Comparator:** no RJ conferences in prison or on probation; community sentence at Magistrates Court.  **Outcomes:** statistically significant reductions in number of offences committed for both intervention and control groups for prison population; no significant differences between intervention and control group for reconviction in terms of costs, rates of recidivism, severity of reconviction, and frequency of reconviction.  **Study Design:** random control trials with cases randomly allocated to either conference or control group; two-year follow-up period.  **Results:** Cost-social benefit ratio of £1.27 per £1 invested. |
| Scheme: JRC Northumbria | **Population:** 206 youth offenders given final warning and 107 adult cases from Magistrates Court; property and violent offences.  **Intervention:** diversion to a police caution with RJC for youth; post guilty plea and pre-sentence for adults.  **Comparator:** diversionary proposal to caution with no RJC for youth; no RJC pre-sentence and sentencing in Magistrates Court for adults.  **Outcomes:** statistically significant reductions in recidivism and severity of reconviction for adult property offenders; no significant reductions in frequency of reconviction or reconvictions in terms of cost.  **Study Design:** random control trials - cases randomly allocated to either conference or control group; two-year follow-up period.  **Results:** Cost-social benefit ratio of £0.59 per £1 spent. |
| Surrey Scheme Evaluation (Mackie et al., 2014) | **Population:** young offenders under 18 (35% under 16); 25% male offenders; not serious offences with theft and handling and violence most common; control group constructed from pre-court and court convictions 2009-11.  **Intervention:** Youth Restorative Intervention; conferencing, direct or indirect mediation; pre-court disposal.  **Comparator:** Alternative to youth caution, youth conditional caution and prosecution.  **Outcomes:** 91% victim satisfaction with involvement; reduction in first time entrants (from 394 in 2010-11 to 189 in 2012-13); lower reoffending rates in intervention group (27%) than control group (33%) with 18% reduction.  **Study Design:** YRI run by Surrey Police and Surrey County Council’s Youth Support Service; default assignment to YRI, control group constructed from previous data.  **Results:** Cost-social benefit ratio of £3.41 for every £1 spent. |
| US Scheme, Economic Evaluation (Furman, 2012) | **Population:** adult offenders (196 RJ and 1,786 conventional justice (CJ) cases) in Massachusetts, including property crimes, substance-related charges, theft/attempted theft charges, and assault/crimes against persons.  **Intervention:** alternative and diversion RJ procedures, sample from Communities for Restorative Justice 2000-9 data.  **Comparator:** ‘traditional’ criminal justice procedures, sample from 2008 Males Release Cohort data.  **Outcomes:** produced cost analysis of RJ, showing net cost savings through reductions in reoffending.  **Study Design:** cost-effectiveness analysis/economic evaluation of RJ using recidivism rates; Massachusetts, US.  **Results:** Cost-social benefit ratio of $3.13 per $1 spent. |

**Abbreviations:** CJ, conventional justice; RJ, restorative justice; RJC, Restorative Justice Congress; YOT, Youth Offender Team; YRI, Youth Restorative Intervention.

# Rates of attrition

Table 3 presents different estimates for the rates of attrition throughout Restorative Justice pathways.

**Table 3: Rates of attrition during the Restorative Justice pathway**

| **Source** | **Cases that drop out at the Referral stage** | **Cases that drop out at the Assessment and Consent stage** | **Cases that engage in an indirect intervention** | **Cases that engage in a direct intervention** |
| --- | --- | --- | --- | --- |
| Police Force Area 1 (Expert opinion) | 20% | 20% | 15% | 45% |
| Police Force Area 2 (Data, not on file), 2021 | 41% | 40% | 6% | 13% |
| Police Force Area 3 (Data on file)*, 2021 | n/a** | 33.4% | 8.2% | 58.4% |
| Provider 1 (Data on file), multiple years | 46% | 46% | 6% | 2% |
| Pathfinder study, 2014-2015 (Kirby & Jacobson, 2015) | 79%** | n/a | 12% | 9% |
| Shapland JRC Phase 1, 2001-2002 (Shapland et al., 2006) | n/a*** | 65% | 0% | 35% |
| Shapland JRC Phase 2, 2002-2003 (Shapland et al., 2006) | n/a** | 71% | 0% | 29% |

**Note:** these figures are exploratory and should be used with caution. *Estimated by comparing referrals to interventions over a two-year period, meaning estimates should be used with caution. **Referral and assessment stage combined. ***These studies followed a different model, where offenders were proactively identified at different stages in the criminal justice process. These figures are a weighted average across the included JRC trials.

# Transforming the treatment effect in Strang 2013

The reduction in reoffending associated with Restorative Justice from the Strang 2013 meta-analysis is presented in Table 4.

Table 4: Strang 2013 meta-analysis of studies assessing the reduction in frequency of reoffending associated with Restorative Justice when delivered as a supplement or substitute to conventional justice, 2 years follow up

| **Group** | **Study** | **Offence** | **Jurisdiction** | **Population** | **Standard mean difference** | **p-value** |
| --- | --- | --- | --- | --- | --- | --- |
| Substitute | JPP | Property | Australia | Youth | 0.137 | 0.283 |
|  | JVC | Violence | Australia | Youth | -0.279 | 0.126 |
|  | *Subgroup* |  |  |  | *-0.051* | *0.807* |
| Supplement | NFW | Juvenile | UK | Youth | -0.276 | 0.048 |
|  | TVP | Violence | UK | Adults | -0.144 | 0.488 |
|  | LOR | Violence | UK | Adults | -0.044 | 0.838 |
|  | IND | Juvenile | USA | Youth | -0.2 | 0.005 |
|  | NCP | Property | UK | Adults | -0.201 | 0.426 |
|  | LOB | Property | UK | Adults | -0.105 | 0.497 |
|  | NCA | Violence | UK | Adults | -0.333 | 0.273 |
|  | TVC | Violence | UK | Adults | -0.247 | 0.326 |
|  | *Subgroup* |  |  |  | *-0.193* | *0.000* |
| **Overall** | **Overall** |  |  |  | **-0.185** | **0.000** |

**Source:** (Strang et al., 2013), **Notes:** A key for the study abbreviations is included in the appendix

The relevant treatment effect was transformed from a standardised mean difference to a rate ratio, using equations from the Cochrane Handbook (Higgins & Green, 2011; Strang et al., 2013)(Equation 1). This equation is for odds ratios and the Strang 2013 study describes the values as odds ratios. However, within the Strang 2013 study, and within this study, reoffending is being analysed as a rate, rather than a dichotomous outcome. These treatment effects are therefore best described as rate ratios.

Equation 1: Using a standardised mean difference to estimate an odds ratio

1. $lnOR=\frac{\pi}{\sqrt{3}}SMD$

# Multiplier 2

The estimates for Multiplier 2 were validated by estimating the implied proportion of total crime that would be committed by reoffenders. In general, the results were plausible. For thefts, the modelled values for Multiplier 2 implied that the majority of ‘thefts from the person’ committed are by reoffenders in their first year. This seemed implausibly high. A thorough investigation was conducted, which yielded the following findings:

- - The total number of police recorded crimes varies modestly year-on-year, without a clear trend. The same is true at the offence group level, but with much more substantial variation year-on-year.
  - The number of proven offences, and therefore the proportion of offences proven, is falling substantially year-on-year.
  - The fact that the components of M2 vary over time implies that M2 is not consistent over time.
  - Time lapses between a crime occurring, being reported, and being proven. This means that estimates for these variables from annual government data sets will not be aligned, because a crime could occur in one year but be proven in the next year and thus be reported in two separate annual returns.
  - This method assumes that the proportion of crimes that are proven is the same as the proportion of reoffences that are proven. It may be that reoffences are more likely to be proven, because they are committed by individuals known to the criminal justice system.
  - The Pro Bono Economics note calculated a multiplier of 15.1 pooled across all crime types, excluding fraud and crimes against society. This is largely aligned with the offences included within this analysis. (Pro Bono Economics, 2019).
  - A multiplier of 19.1 for thefts implies that approximately 5% of thefts reported to the police result in a proven outcome. This estimate is plausible given the high proportion of thefts that are not investigated.

# Estimating the cost of Restorative Justice

There are three possible approaches to estimating the cost of Restorative Justice: ‘top down’, ‘bottom up’ and ‘market research’.

### Top down

Adopting a ‘top down’ approach means estimating the total cost of delivering Restorative Justice interventions and total number of Restorative Justice interventions delivered within a particular locality, then dividing the former by the latter.

### Bottom up

A ‘bottom up’ approach requires taking a ‘dip sample’ of Restorative Justice cases, tracking all their contacts with a Restorative Justice service, then assigning a cost to each of these contacts. Some additional work is required to apportion any fixed costs, or overheads, such as facilities and management costs.

### Market research

A ‘market research’ approach requires collecting data from a sample of Restorative Justice services on the price they would charge to provide a Restorative Justice intervention to a typical case or group of cases.

### Options appraisal for costing Restorative Justice

There were a number of challenges with a ‘top down’ approach. We were not able to access the required data and they are not routinely published. Experts advised that contracts Police and Crime Commissioners agree with Restorative Justice services rarely specify a defined capacity, or a defined number of staff, and data on either of these measures of activity are rarely published. Instead, contracts tend to specify a requirement to provide Restorative Justice interventions for a defined locality, known as a capitated payment. The Why me? report series, Valuing Victims, presents data on Restorative Justice activity and spending, accessed via a Freedom of Information (FOI) request from the Ministry of Justice (Why me?, 2021). The Valuing Victims report makes clear that issues with missing data and poor data quality mean that these data cannot be relied upon for analyses.

A ‘market research’ approach was also challenging. As stated above, Restorative Justice interventions are mostly commissioned for defined localities, not on a case-by-case, or block purchase (fixed quantity) basis. We did receive some estimates for providing Restorative Justice interventions on a case-by-case basis. These estimates varied substantially (from £250 to £1,150) (data on file), and it was not clear exactly how the interventions, or eligible cases, were defined so it was challenging to determine the relevance of these estimates to this analysis.

For these reasons, this research primarily used ‘bottom up’ estimates of the cost of delivering Restorative Justice interventions. Other cost estimates were used within sensitivity analysis.

The time taken to perform different steps of the Restorative Justice process was estimated using data and expert insight provided by those involved in managing Restorative Justice services. Expert insight from the Restorative Justice Coordinator at Why me?, and others, were used to supplement the data and incorporate additional costs borne by the Restorative Justice service (Why me?), the referrer, and other agencies.

### Costing Restorative Justice

Resource use estimates at the different stages in the Restorative Justice pathway were provided by Restorative Justice services (Table 5). These were subject to substantial uncertainty because costs vary between cases and the number of cases that progress to a direct Restorative Justice intervention in a year is often relatively low for each Restorative Justice service.

**Table 5: Resource use per case for Restorative Justice provider**

| **Source** | **Referral** | **Assessment** | **Direct Restorative Justice** | **Indirect Restorative Justice** |
| --- | --- | --- | --- | --- |
| Police Force Area 1 (Data on file) | 45 minutes | 1,100 hours* | No data | No data |
| Police Force Area 2 (Data not on file) | 45 minutes | 7 hours | 79 hours | 19 hours |
| Provider 1 (Data on file) | 30 minutes | 7 hours | No data | 28 hours |
| Youth Offending Team 1 (expert opinion) | 30 minutes | 7 hours | 10 hours | No data |
| Pathfinder** (Kirby & Jacobson, 2015) | 30 minutes | 7 hours | 123 hours | 68 hours |

**Note:** These figures are exploratory and should be used with caution. *This estimate is skewed by a small number of cases with exceptionally high resource use. It was not possible to fully establish the robustness of this estimate. **Total estimates are given (including direct and indirect), no further breakdown is reported. This estimate approximates apportioning this total according to the stages in the Restorative Justice pathway.

Base case estimates are presented in Table 6, informed by expert opinion on which resource use estimates are likely to be most representative of current practice. These estimates are subject to substantial uncertainty and there is substantial variation between cases. In particular, a number of agencies spent disproportionate time and resources on a small number of highly complex cases that often remained open for several years.

For this cohort, experts advised that most referrals are self-referrals by the victim. Further, for the majority of cases, agency input is limited to the prison service, or probation service, facilitating contact with the offender. However, this can vary, with some cases involving more substantial input from other agencies.

In the base case, it was assumed that the majority of input relates to the Restorative Justice service. A scenario was implemented with the cost of the Restorative Justice process increased by 50%, to account for this uncertainty.

**Table 6: Base case resource use estimates for the Restorative Justice pathway**

| **Source** | **Inputs** | **Notes** |
| --- | --- | --- |
| **Referral** | 45 minutes of Restorative Justice worker/volunteer time. | Restorative Justice worker time informed by data and expert opinion. Most referrals for this cohort are self-referrals by the victim. |
| **Assessment** | 15 minutes of police constable time.  7 hours Restorative Justice worker/volunteer time.  1 prison visit. | Police constable time to help locate the other party, informed by expert opinion.  Restorative Justice worker time. |
| **Direct Restorative Justice** | 79 hours of Restorative Justice worker time. | Informed by Police Force Area 2 data. |
| **Indirect Restorative Justice** | 19 hours of Restorative Justice worker time. | Informed by Police Force Area 2 data. |
| **Additional costs** | Restorative Justice manager time is 20% of Restorative Justice worker time, at every stage. | Informed by expert opinion. |

The base case resource use estimates (Table 6) were combined with unit costs from published sources (Table 7), to model the costs of each stage of the Restorative Justice pathway. These combined estimates are presented in the paper.

**Table 7: Unit costs of inputs to the Restorative Justice pathway**

| **Item** | **Cost** | **Year** | **2021 cost** | **Notes** | **Source** |
| --- | --- | --- | --- | --- | --- |
| 1 hour Restorative Justice worker | £12.79 | 2021 | £12.79 | £8.91 national minimum wage and 44% additional oncosts. | (Gov.uk, n.d.)  (ASFB: Accounting Services for Businesses, n.d.) |
| 1 hour Restorative Justice Manager | £29 | 2009 | £36.81 | Based on a case manager/Youth Offending Team practitioner salary | (Brookes et al., 2013) |
| 1 hour police constable | £58.99 | 2017 | £65.83 |  | (The National Police Chiefs’ Council, 2019) |
| 1 hour Probation officer time | £29 | 2009 | £36.81 |  | (Brookes et al., 2013) |
| 1 hour prison officer time | £29 | 2009 | £36.81 | Assumed to align with probation officer/YOT Team practitioner, given similarity in salaries. | See above |

# References

ASFB: Accounting Services for Businesses. (n.d.). *True Cost of an Employee Calculator*. Retrieved August 2, 2022, from https://www.accountingservicesforbusiness.co.uk/calculators1/true-cost-of-an-employee

Brookes, N., Barrett, B., Netten, A., & Knapp, E. (2013). *Unit Costs in Criminal Justice (UCCJ)*. www.pssru.ac.uk

Furman, J. M. (2012). *An Economic Analysis of Restorative Justice*. http://www.antoniocasella.eu/restorative/Furman_2012.pdf

Gov.uk. (n.d.). *National Minimum Wage and National Living Wage rates*. Retrieved August 2, 2022, from https://www.gov.uk/national-minimum-wage-rates

Higgins, J., & Green, S. (2011, March). *Cochrane Handbook for Systematic Reviews of Interventions* (J. Higgins & S. Green, Eds.). The Cochrane Collaboration. https://handbook-5-1.cochrane.org/chapter_9/9_2_3_2_the_standardized_mean_difference.htm

Kirby, A., & Jacobson, J. (2015). *Evaluation of the Pre-Sentence RJ Pathfinder*.

Macdonald, M., Williams, J., Kane, D., & Rowe, S. G. (2017). *The Restorative Justice Project: Evaluation of Restorative Justice Conferences*. https://www.open-access.bcu.ac.uk/6453/

Mackie, A., Cattell, J., Reeder, N., & Webb, S. (2014). *Youth Restorative Intervention Evaluation Final report*.

Matrix Evidence. (2009). *Economic analysis of interventions for young adult offenders*. https://barrowcadbury.org.uk/wp-content/uploads/2011/01/Matrix_Economic_analysis-T2A-2009.pdf

Pro Bono Economics. (2019). *Reducing Reoffending Methodology note*. https://whatworks.college.police.uk/Pages/default.aspx

Shapland, J., Atkinson, A., Atkinson, H., Chapman, B., Colledge, E., Dignan, J., Howes, M., Johnstone, J., Robinson, G., & Sorsby, A. (2006). *Restorative justice in practice: the second report from the evaluation of three schemes*. Centre for Criminological Research, University of Sheffield. https://restorativejustice.org.uk/sites/default/files/resources/files/Full%20report.pdf

Shapland, J., Atkinson, A., Atkinson, H., Dignan, J., Edwards, L., Hibbert, J., Howes, M., Johnstone, J., Robinson, G., & Sorsby, A. (2008). *Does restorative justice affect reconviction?: the fourth report the evaluation of three schemes*. National Offender Management Service. https://restorativejustice.org.uk/sites/default/files/resources/files/Does%20restorative%20justice%20affect%20reconviction.pdf

Shewan, G. (2010). *A Business Case for Restorative Justice and Policing*.

Soppitt, S., & Irving, A. (2011). *An Evaluation into the Effectiveness of the Early Diversion Intervention “Triage” as a Mechanism for Tackling Youth Offending in Newcastle Upon Tyne*.

Strang, H., Sherman, L. W., Mayo‐Wilson, E., Woods, D., & Ariel, B. (2013). Restorative Justice Conferencing (RJC) Using Face‐to‐Face Meetings of Offenders and Victims: Effects on Offender Recidivism and Victim Satisfaction. A Systematic Review. *Campbell Systematic Reviews*, *9*(1), 1–59.

The National Police Chiefs’ Council. (2019). *National Policing Guidelines on Charging for Police Services*.

Why me? (2021). *Valuing Victims: A Review of Police and Crime Commissioners’ Delivery of Restorative Justice 2019/20*.
